# Paclobutrazol Enhanced Stem Lodging Resistance of Direct-Seeded Rice by Affecting Basal Internode Development

**DOI:** 10.3390/plants13162289

**Published:** 2024-08-17

**Authors:** Weiyang Liu, Jiehao Cui, Cheng Ran, Yuchen Zhang, Jianuo Liang, Xiwen Shao, Qiang Zhang, Yanqiu Geng, Liying Guo

**Affiliations:** 1 Agronomy College, Jilin Provincial Laboratory of Crop Germplasm Resources Jilin Agricultural University, Changchun 130118, China; liuweiyang1552@126.com (W.L.); cuijiehao0626@163.com (J.C.); jlndrc@126.com (C.R.); 18043814851@163.com (Y.Z.); 18233176551@163.com (J.L.); shaoxiwen@126.com (X.S.); qiangz@jlau.edu.cn (Q.Z.); 2 Heyuan Branch, Guangdong Laboratory for Lingnan Modern Agriculture, Heyuan 517000, China; 3 National Crop Variety Approval and Characterization Station, Jilin Agricultural University, Changchun 130118, China

**Keywords:** direct-seeded rice, lodging resistance, paclobutrazol, structural carbohydrate, endogenous hormones

## Abstract

The objectives of this study were to explore the mechanism of stem mechanical strength in direct-seeded rice (DSR) as affected by paclobutrazol, especially its related endogenous hormone and cell wall component changes in culm tissue and response to the application of paclobutrazol. Field experiments were conducted in Changchun County, Jilin Province, China, by using two japonica rice varieties, Jiyujing and Jijing305, with soaking seeds in paclobutrazol at concentrations of (0 mg L^−1^, S0; 50 mg L^−1^; S1; 100 mg L^−1^; S2; 150 mg L^−1^, S3; 200 mg L^−1^, S4) in 2021 and 2022. The results suggest that the application of paclobutrazol increased the grain yield and reduced the lodging rate of DSR. Compared with the S0 treatments, soaking the seeds in paclobutrazol treatments rapidly shortened the length of the basal internode by decreasing the endogenous indole acetic acid (IAA) and gibberellin A3 (GA_3_) contents in culm tissue. The larger breaking strength (M) was attributed to a higher section modulus (SM) and bending stress (BS). The higher mechanical tissue thickness in culm tissue under paclobutrazol treatments, which was raised by higher endogenous zeatin and zeatin riboside (Z+ZR) content in culm tissue, increased the culm diameter, culm wall thickness, and section modulus (SM) of the internode. Compared with the S0 treatments, soaking the seeds in paclobutrazol treatments increased the cellulose content, lignin content, activities of lignin-related enzymes, and expression of key genes in lignin biosynthesis, as well as resulted in a higher bending stress (BS) to enhance the culm breaking strength (M).

## 1. Introduction

Rice (*Oryza sativa* L.) is one of the most cultivated cereals globally and a staple food crop for billions of people. In China, rice is the second-largest cereal crop, and accounts for 32% of the overall grain production; over 50% of the population relies on rice as a primary food source [1]. Rice yield in China plays an essential role in ensuring food security [2]. Conventional transplanted rice requires extensive labor input, energy, and substantial irrigation or rainfall during land preparation and growth [3]. Therefore, in the upcoming decades, it would be wise to shift from conventional transplanted rice to other systems that enhance water efficiency without compromising crop yield, ultimately promoting environmental health sustainability.

Direct-seeded rice (DSR) is a rice cultivation system that refers to the process of establishing a rice crop from seeds sown directly in the field as opposed to transplanting seedlings from the nursery [4]. Compared with conventional transplanted rice, DSR offers benefits such as improved water use efficiency [5], suitability for mechanization [4], higher economic returns, and maintaining yield levels [6]. A growing number of traditional transplanted rice has been converted to DSR [7], with rice being cultivated using either a direct-seeded system in the United States, Australia, and Europe [8]. The area dedicated to DSR in South Korea accounts for 50% or more of the country’s rice planting area, and numerous Southeast Asian countries have begun to adopt DSR farming practices as well [1,9], indicating that the yield stability and potential of DSR have become progressively vital for maintaining food security.

Recently, due to the increased frequency of various weather phenomena, such as high wind speeds, lodging has emerged as a significant factor limiting rice yield. Lodging leads to a decrease in yield by diminishing the photosynthesis of the canopy [10], hindering the movement of assimilates [11], and triggering fungal infections [12]. The stress caused by lodging during the grain-filling phase has led to a loss in yield ranging from 2.66 to 2.71% [13]. A commonly used practice for direct-seeded rice is a high seeding rate, aimed at minimizing yield loss due to inadequate crop establishment. However, a high seeding rate causes overluxuriant growth that leads to tall plants and a thin culm and ultimately reduces lodging resistance [4]. Therefore, DSR results in a higher lodging risk than conventional transplanted rice [14]. Therefore, comprehensively understanding the processes influencing the lodging of DSR is crucial for mitigating the vulnerability of DSR production to severe weather events.

There are two types of lodging in cereal plants: stem or stalk lodging (stem-breaking type and stem-bending type) and root lodging. In China, the occurrence of stem lodging is more prevalent [15]. Many researchers have reported that stem lodging mainly occurs at the basal internode [15,16,17]. The breaking strength of the basal internode determines rice plant susceptibility to lodging [17,18]. Consequently, enhancing the stiffness of the basal stem has emerged as a key research focus aimed at improving crop lodging resistance and boosting grain yield.

Studies have indicated that the culm diameter, filling degree, wall thickness, and stiffness rigidity play significant roles in stem mechanical strength or lodging resistance [15,19]. The relationship between the mechanical strength of the basal internodes and the nonstructural and structural carbohydrate contents of the internode is significant [20]. Many studies have revealed that, as primary structural elements of the secondary cell wall, cellulose and lignin are intricately linked to the mechanical strength of the culm [15,21,22]. Enhancing culm strength by increasing lignin accumulation is also a new target to reduce lodging stress in crops [22,23]. Furthermore, the breaking strength of basal internodes is related to morphological characteristics and anatomical characteristics [11,24]. The mechanical strength of the crop stem is closely linked to anatomical characteristics such as mechanical tissue, the number and area of vascular bundles, and the cross-sectional area [25]. Vascular bundles play an essential role in the lodging resistance of rice stems, with their number and cross-sectional area being determining factors [26]. Cell units are integral to stem anatomy, and the processes of cell elongation and division are influenced by endogenous hormones, impacting internode length and plant structure [19]. However, it remains unclear how changes in developmental mechanisms at the endogenous hormones affect internode elongation and mechanical strength and their relationship with stem lodging resistance in DSR.

Plant growth regulators have been widely used to modify hormonal balance and improve the physiological traits of crops. The use of plant growth regulators quickly alters the height and stem characteristics of plants, consequently enhancing lodging resistance and increasing yield in cereal crops [16,27,28,29]. Paclobutrazol [(2RS,3RS)-1-(4-chlorophenyl)-4,4-dimethyl-2-(1H-1,2,4-TRIzol-1-yl)-pentan-3-ol] is a member of the triazole family with growth-regulating properties and is widely used in agriculture. Previous studies have revealed that paclobutrazol can increase the growth of crop stems, reduce the length of the basal internode, reduce plant height, enhance the mechanical properties of stems, and significantly enhance the lodging resistance of crops [15,30,31,32,33]. The application of paclobutrazol could increase the lignin content and lignin-related enzyme activities in maize and wheat [15,34]. However, information on the effects of paclobutrazol on the lodging resistance of DSR stems is limited, and the ways in which it impacts physical strength, stem morphological characteristics, and the lignin accumulation of DSR still remains inadequately understood.

Consequently, the objective of the present study was to (i) explore the effects of paclobutrazol application on the physical strength, morphological and anatomical characteristics, and carbohydrate content of the DSR stem and their relationship with lodging resistance, and (ii) determine the relationship between internode-related morphological traits and endogenous hormones, providing guidelines for cultivation practices that enhance grain yield while improving lodging resistance in DSR. 

## 2. Results

### 2.1. Yield and Lodging Rate 

**Figure 1:**
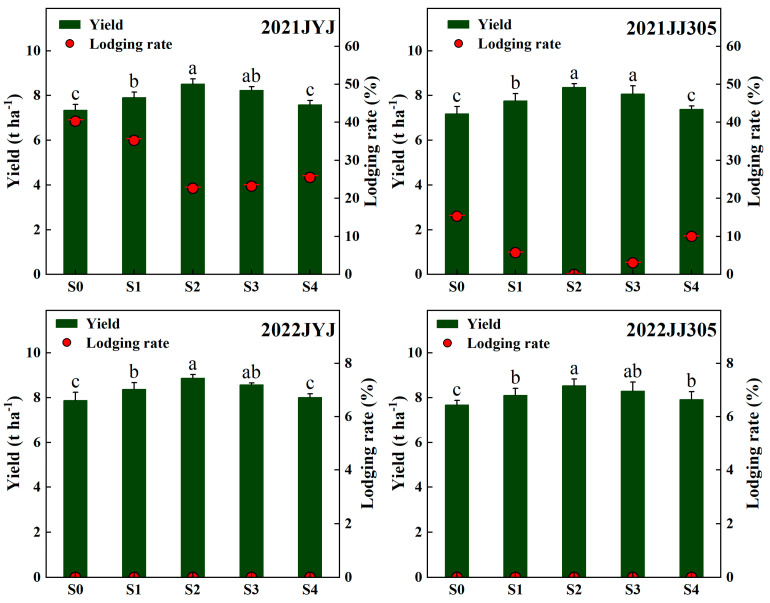
Effects of paclobutrazol application on the grain yield and lodging rate of DSR in 2021 and 2022. S0, S1, S2, S3, and S4 represent seed soaking with paclobutrazol at concentration of 0, 50, 100, 150, and 200 mg L^−1^, respectively. The columns mean the yield and the points represent the lodging rate. Different letters indicate significant differences between different paclobutrazol treatments (*p* < 0.05, Tukey’s test). Bars represent the standard deviation of the mean (*n* = 3).

Soaking the seeds in paclobutrazol reduced the lodging rate but increased the grain yield (Figure 1). There were no lodging events occurring at the experimental region in 2022; therefore, the lodging rate was only analyzed in 2021. As the concentration of paclobutrazol increased, the yield of direct-seeded rice (DSR) first increased and then decreased, while the lodging rate first decreased and then increased. The S2 treatment achieved a higher yield and lower lodging rate. Compared with the S0 treatment, the S2 treatment reduced the lodging rate of JYJ by 17.7% (2021) and enhanced the yield by 14.12% (means based on 2021–2022); decreased the lodging rate of JJ305 by 10.0% (2021) and enhanced the yield by 13.79% (means based on 2021–2022). The above results indicate that seed soaking with paclobutrazol increases the yield and reduces the lodging rate of DSR (Figure 1).

### 2.2. Internode Length

**Figure 2:**
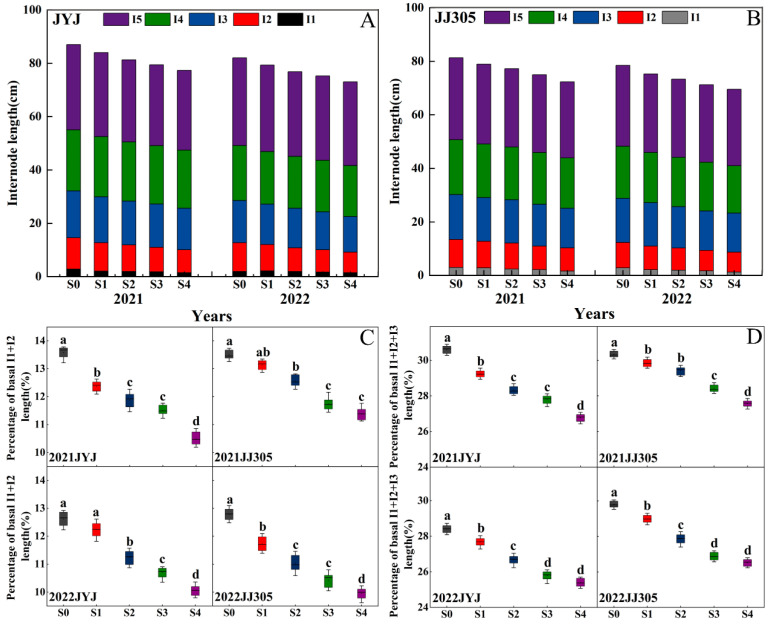
(**A**,**B**) The length of the 1st (I1), 2nd (I2), 3rd (I3), 4th (I4), and 5th (I5) internodes, which are numbers from the bottom of the culm in JYJ (**A**) and JJ305 (**B**) in 2021 and 2022. (**C**) The percentage of the total internode length made up of basal internodes (I1+ I2) in two rice cultivars JYJ and JJ305 in 2021 and 2022. (**D**) The percentage of the total internode length made up of basal internodes (I1+ I2 + I3) in two rice cultivars JYJ and JJ305 in 2021 and 2022. S0, S1, S2, S3, and S4 represent seed soaking with paclobutrazol at concentration of 0, 50, 100, 150, and 200 mg L^−1^, respectively. Different letters indicate significant differences between different paclobutrazol treatments (*p* < 0.05, Tukey’s test). Bars represent the standard deviation of the mean (*n* = 8).

Compared to the S0 treatment, soaking the seeds in paclobutrazol treatments reduced the plant height of DSR (Figure 2A,B). Increasing the concentration of paclobutrazol from low to high levels significantly lowered the lengths of I1, I2, I3, I4, and I5 in both varieties and years. Compared to the S0 treatment, the S1, S2, S3, and S4 treatments decreased the second basal internode length of JYJ and JJ305 by 8.61% and 6.21%, 12.26% and 10.21%, 18.84% and 15.83%, and 22.82% and 19.64%, respectively (means based on two years) (Figure 2A,B). Further analysis of the proportion of basal internodes in the total internode length revealed that paclobutrazol application resulted in a decrease in the percentage of the total internode length made up of the basal internodes (I1 + I2 and I1 + I2 + I3) (Figure 2C,D).

### 2.3. Endogenous Hormones in the Culm

**Figure 3:**
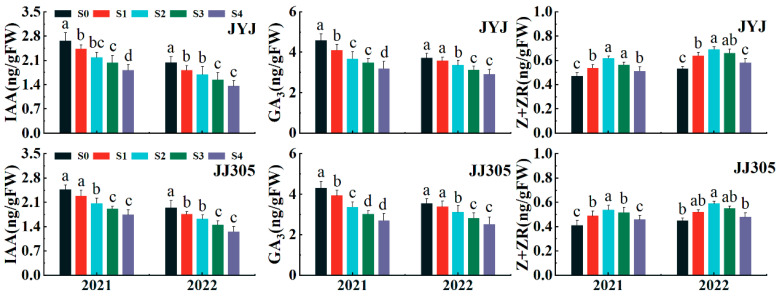
Effects of paclobutrazol application on the endogenous hormone of the second basal internode of DSR in 2021–2022. IAA: indole acetic acid; GA_3_: gibberellin A3; Z+ZR: zeatin and zeatin riboside. S0, S1, S2, S3, and S4 represent seed soaking with paclobutrazol at concentration of 0, 50, 100, 150, and 200 mg L^−1^, respectively. Different letters indicate significant differences between different paclobutrazol treatments (*p* < 0.05, Tukey’s test). Bars represent ± SD (*n* = 3).

The endogenous hormone contents, including indoleacetic acid (IAA), gibberellin 3 (GA_3_), and zeatin and zeatin riboside (Z+ZR), were closely related to cell division and cell elongation in culm tissue. The endogenous hormones in the culm were influenced significantly by paclobutrazol application. Increasing the concentration of paclobutrazol from low to high levels significantly reduced the IAA and GA_3_ contents of the culm (Figure 3). Where paclobutrazol application increased the Z+ZR content of the culm, the culm Z+ZR content first increased and then decreased with increasing the concentration of paclobutrazol from low to high levels, and the maximum Z+ZR content was obtained with the S2 treatment. 

### 2.4. The Culm Diameter, Culm Wall Thickness, and Culm Filling Degree

**Figure 4:**
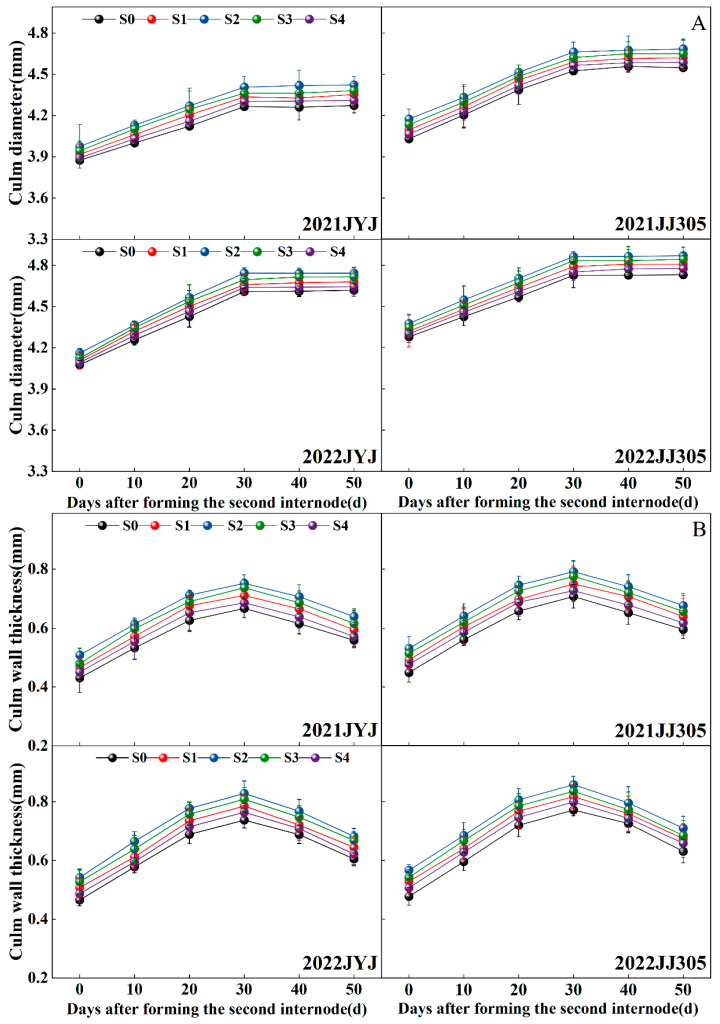

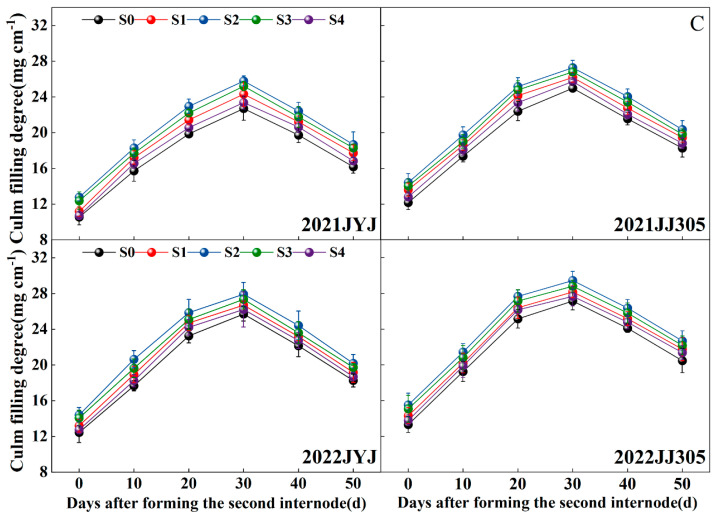
Effects of paclobutrazol application on the culm diameter (**A**), culm wall thickness (**B**), and culm filling degree (**C**) of the second basal internode of direct-seeded rice from 0d after forming the second internode (DAF0d) to 50d after forming the second internode (DAF50d). S0, S1, S2, S3, and S4 represent seed soaking with paclobutrazol at concentration of 0, 50, 100, 150, and 200 mg L^−1^, respectively. The vertical bars represent the standard error of the mean (*n* = 8).

With the progression of growth, the culm diameter first rapidly increased from 0d (DAF0d) to 30d after the formation of the basal second internode (DAF30d), and then smoothed (Figure 4A). The culm wall thickness and filling degree of the internode first increased from DAF0d to DAF30d, then decreased, and finally peaked on DAF30d (Figure 4B,C). Paclobutrazol application significantly increased the culm diameter, culm wall thickness, and culm filling degree. With increasing concentrations of paclobutrazol from low to high levels, the culm diameter, culm wall thickness, and culm filling degree first increased and then decreased, where the maximum culm diameter and the culm wall thickness of the second basal internode were obtained with the S2 treatments.

Compared with the S0 treatment, the S1, S2, S3, and S4 treatments increased the culm diameter of JYJ and JJ305 by 1.70% and 1.58%, 3.35% and 3.11%, 2.41% and 2.36%, and 0.80% and 0.82%, respectively (means based on two years and six stages) (Figure 4A); increased the culm wall thickness of JYJ and JJ305 by 6.99% and 7.29%, 14.13% and 13.65%, 10.63% and 10.32%, and 3.55% and 4.29%, respectively (means based on two years and six stages) (Figure 4B); increased the culm filling degree of JYJ and JJ305 by 7.02% and 7.45%, 14.90% and 14.28%, 11.22% and 11.03%, and 2.64% and 4.22%, respectively (means based on two years and six stages) (Figure 4C).

### 2.5. Culm Physical Parameters (Breaking Strength, Bending Stress, and Cross-Section Modulus)

**Figure 5:**
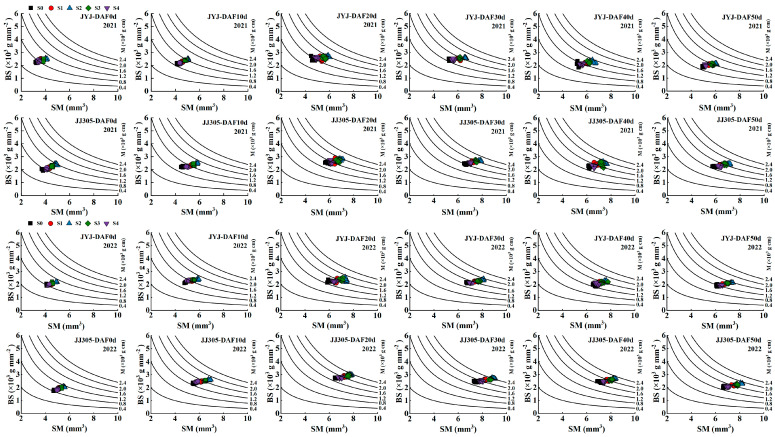
Relationship between bending stress (BS) and section modulus (SM) from 0d after forming the second internode and 50d after forming the second internode in 2021–2022, respectively. Curved lines indicate the breaking strength (M). S0, S1, S2, S3, and S4 represent seed soaking with paclobutrazol at concentration of 0, 50, 100,150, and 200 mg L^−1^, respectively. DAF0d, DAF10d, DAF20d, DAF30d, DAF40d, and DAF50d are mean from 0 days after forming the second internode and 50 days after forming the second internode.

When comparing the breaking strength (M) under soaking the seeds in paclobutrazol treatments, we found that the M of DSR first increased from DAF0d to DAF30d, then reduced, and finally peaked on DAF30d (Figure 5). Paclobutrazol application increased the M. With increasing concentrations of paclobutrazol from low to high levels, the M first increased and then decreased, where the maximum M was obtained with the S2 treatments (Figure 5).

Compared with the S0 treatment, the S1, S2, S3, and S4 treatments significantly increased the M of JYJ and JJ305 by 16.50% and 17.09%, 34.47% and 34.35%, 24.21% and 25.10%, and 7.61% and 8.05%, respectively (means based on two years and six stages) (Figure 5), which resulted from a higher bending stress (BS) and section modulus (SM).

M can be further divided into two parameters: SM and BS. The SM of the culm is significantly affected by its morphological characteristics and is correlated with the physical structure of the internodes. Paclobutrazol application increased the SM. With increasing the concentration of paclobutrazol from low to high levels, the SM first increased and then decreased, where the maximum SM was obtained with the S2 treatments. Compared with the S0 treatment, the S1, S2, S3, and S4 treatments increased the culm SM of JYJ and JJ305 by 10.24% and 9.75%, 20.24% and 18.85%, 14.39% and 14.33%, and 4.61% and 5.34%, respectively (means based on two years and six stages) (Figure 5). 

The BS is significantly affected by its physical characteristics. Paclobutrazol application increased the BS of the culm. Increasing the concentration of paclobutrazol from low to high levels, the BS of the culm first increased and then decreased, where the maximum BS of the culm was obtained with the S2 treatments. Compared with the S0 treatment, the S1, S2, S3, and S4 treatments increased the culm BS of JYJ and JJ305 by 5.49% and 4.32%, 9.93% and 8.88%, 7.32% and 6.01%, and 1.63% and 0.27%, respectively (means based on two years and six stages) (Figure 5).

### 2.6. Lignin Content and Lignin-Related Enzymes Activities of Culm

#### 2.6.1. Lignin Content

**Figure 6:**
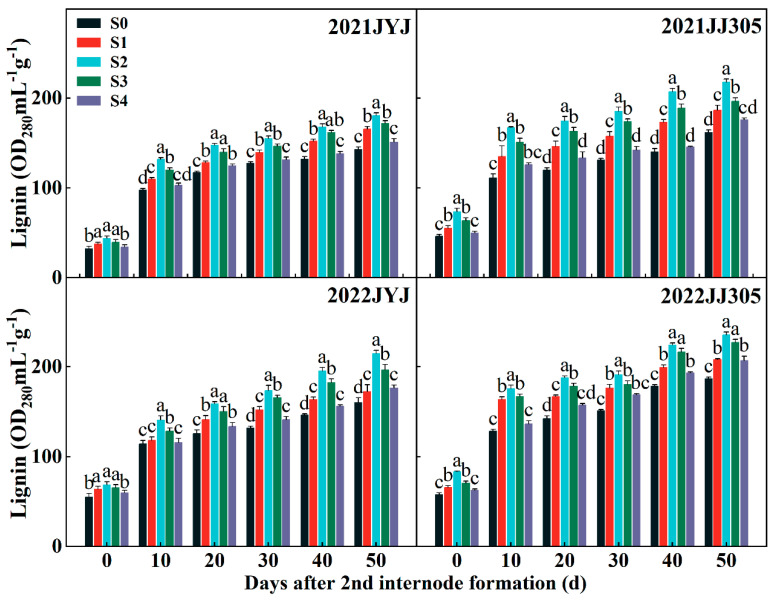
Effects of paclobutrazol application on the lignin content of the second basal internode of direct-seeded rice. S0, S1, S2, S3, and S4 represent seed soaking with paclobutrazol at concentration of 0, 50, 100, 150, and 200 mg L^−1^, respectively. Different letters indicate significant differences between different paclobutrazol treatments (*p* < 0.05, Tukey’s test). Bars represent the standard deviation of the mean (*n* = 3).

The lignin content increased gradually from DAF0d to DAF50d under soaking the seeds in paclobutrazol treatments. Upon increasing the paclobutrazol concentration from low to high levels, the accumulation of lignin first increased and then decreased, whereas the S2 treatments obtained a higher lignin content. Compared with the S0 treatment, the S1, S2, S3, and S4 treatments increased the culm lignin content of JYJ and JJ305 by 12.01% and 18.23%, 28.58% and 38.59%, 20.39% and 27.81%, and 6.06% and 9.15%, respectively (means based on two years and six stages) (Figure 6). 

#### 2.6.2. Activities of Lignin-Related Enzymes

**Figure 7:**
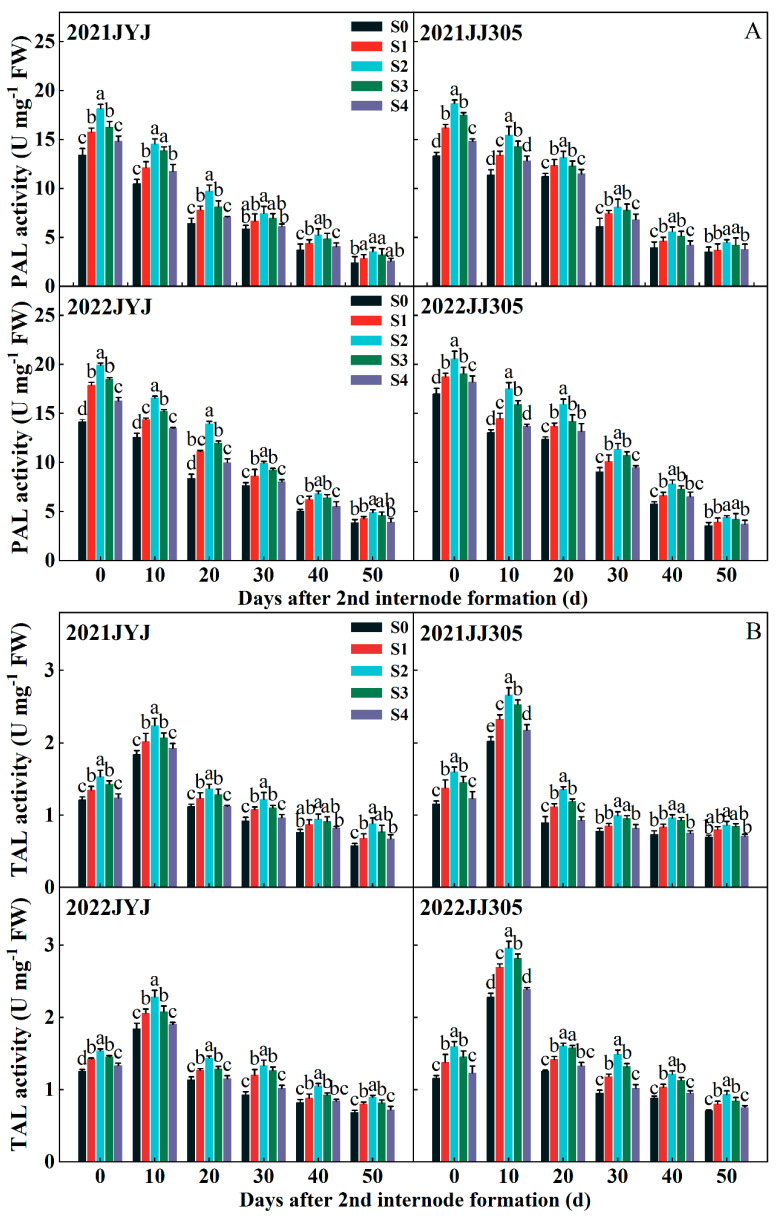

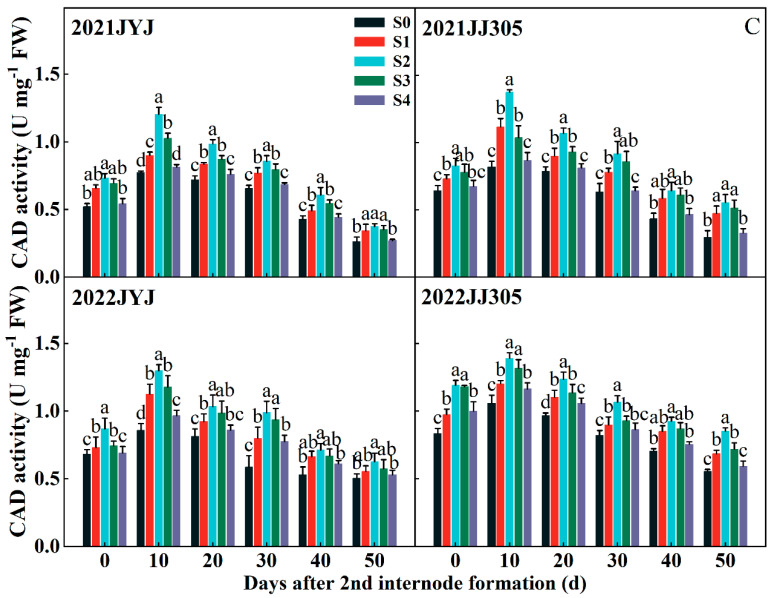
Effects of paclobutrazol application on the PAL activity (**A**), TAL activity (**B**), and CAD activity (**C**) of the second basal internode of direct-seeded rice. S0, S1, S2, S3, and S4 represent seed soaking with paclobutrazol at concentration of 0, 50, 100, 150, and 200 mg L^−1^, respectively. Different letters indicate significant differences between different paclobutrazol treatments (*p* < 0.05, Tukey’s test). Bars represent the standard deviation of the mean (*n* = 3).

The PAL activity enzyme of culm basically decreased from DAF0d to DAF50d under seed soaking with paclobutrazol treatments. With increasing the paclobutrazol concentration from lower to higher levels, the PAL enzyme activity first increased and then decreased, where the S2 treatments obtained higher PAL enzyme activity (Figure 7A). Compared with the S0 treatment, the S2 treatment increased the PAL enzyme activity of the culm of JYJ and JJ305 by 32.65% and 28.52%, respectively (means based on two years and six stages) (Figure 7A).

The TAL enzyme activity of the culm first increased, then decreased from DAF0d to DAF50d, and peaked on DAF10d (Figure 7B). Paclobutrazol application increased TAL enzyme activity. The TAL enzyme activity first increased and then decreased gradually with the application of paclobutrazol from lower to higher concentrations. Compared with the S0 treatment, the S2 treatments significantly increased TAL enzyme activity. The S2 treatment increased the TAL enzyme activity of the culm of JYJ and JJ305 by 30.32% and 33.72%, respectively (means based on two years and six stages) (Figure 7B).

The CAD activity increased gradually from DAF0d to DAF10d and then decreased. Paclobutrazol application increased CAD enzyme activity (Figure 7C). The maximum CAD enzyme activity was obtained with the S2 treatments. Compared with the S0 treatment, the S2 treatment increased the CAD enzyme activity of the culm of JYJ and JJ305 by 35.91% and 34.49%, respectively (means based on two years and six stages) (Figure 7C).

### 2.7. The Cellulose Content 

**Figure 8:**
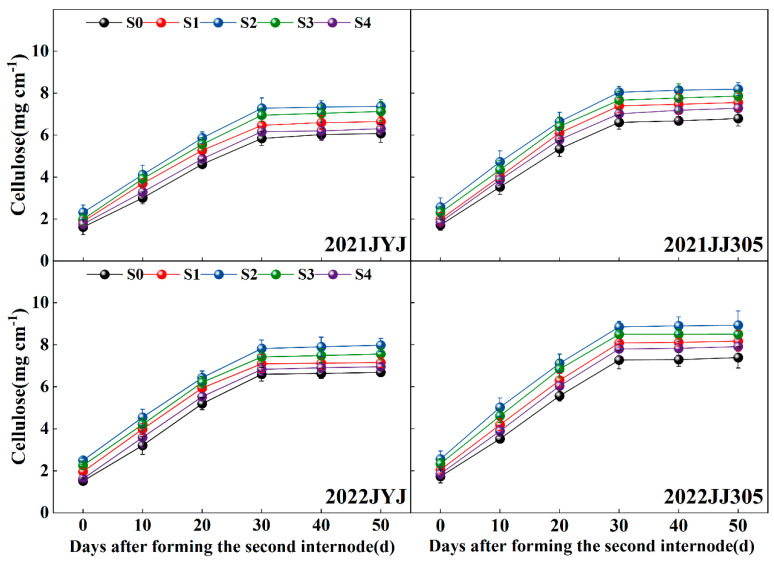
Effects of paclobutrazol application on the cellulose content of the second basal internode of direct-seeded rice. S0, S1, S2, S3, and S4 represent seed soaking with paclobutrazol at concentration of 0, 50, 100, 150, and 200 mg L^−1^, respectively. The vertical bars represent the standard error of the mean (*n* = 3).

With the progression of growth, the cellulose content of the culm first rapidly increased from DAF0d to DAF30d and then smoothed (Figure 8). Paclobutrazol application increased the cellulose content of the culm. With increasing concentrations of paclobutrazol from low to high levels, the cellulose content first increased and then decreased, where the maximum cellulose content was obtained with the S2 treatment (Figure 8). Compared with the S0 treatment, the S1, S2, S3, and S4 treatments increased the culm cellulose content of JYJ and JJ305 by 11.94% and 12.63%, 25.33% and 25.60%, 18.77% and 19.25%, and 5.27% and 7.69%, respectively (means based on two years and six stages) (Figure 8).

### 2.8. The Anatomical Characteristics of Culm Tissue

**Figure 9:**
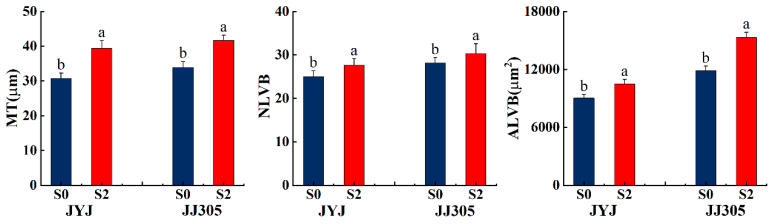
Effects of paclobutrazol application on the anatomical structure of the second basal internode of direct-seeded rice in 2022. MT: mechanical tissue; NLVB: number of large vascular bundles; ALVB: area of large vascular bundles. S0 and S2 represent seed soaking with paclobutrazol at concentration of 0 and 100 mg L^−1^, respectively. Different letters indicate significant differences between different paclobutrazol treatments (*p* < 0.05, Tukey’s test). Bars represent the standard deviation of the mean (*n* = 3).

In order to further explore the relationship between the anatomical structure and structural lodging resistance of DSR and the regulatory effect of paclobutrazol on the anatomical characteristics of DSR stems, the treatment without paclobutrazol (S0) and the treatment of paclobutrazol soaking (S2, with a concentration of 100 mg L^−1^) were selected, the middle section of the second basal internode was taken, and paraffin sectioning was performed at the heading stage. Soaking the seeds in paclobutrazol promoted the development of stem mechanical tissue (Figure 9). Paclobutrazol treatments increased the mechanical tissue thickness and the number and area of large vascular bundles of the basal second internode in DSR stems.

### 2.9. Expression of Genes Involved in Lignin Synthesis 

**Figure 10:**
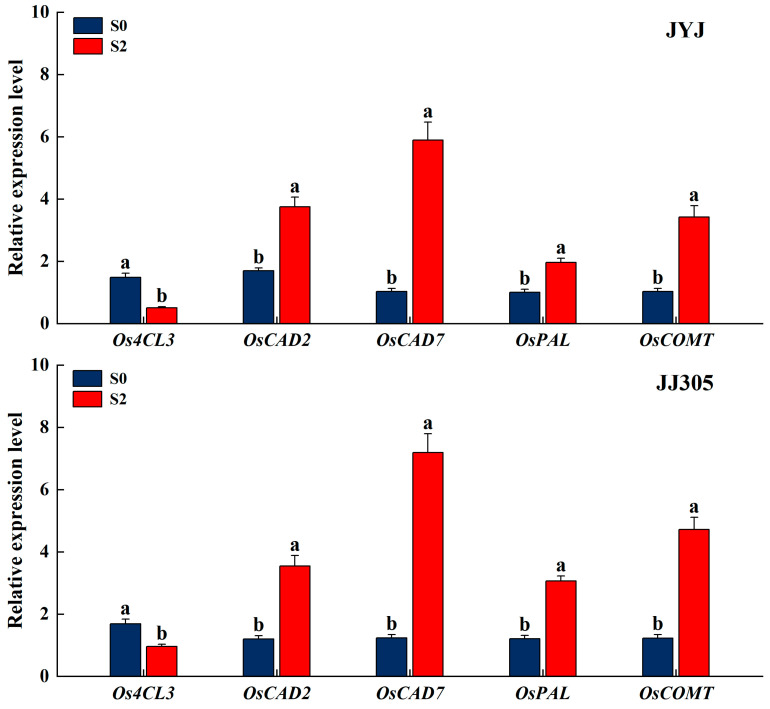
Effects of paclobutrazol application on gene expression of secondary cell wall lignin biosynthesis of the second basal internode of direct-seeded rice in 2022. S0 and S2 represent seed soaking with paclobutrazol at concentration of 0 and 100mg L^−1^, respectively. Different letters indicate significant differences between different paclobutrazol treatments (*p* < 0.05, Tukey’s test). Bars represent the standard deviation of the mean (*n* = 3).

In order to further explore the regulatory effect of paclobutrazol on the biosynthesis of cellulose and lignin with anatomical characteristics of DSR stems, the treatment without paclobutrazol (S0) and the treatment of paclobutrazol soaking (S2, with a concentration of 100 mg L^−1^) were selected, and samples were taken at the heading stage. The expression levels of genes associated with the cellulose and lignin synthesis of the second basal internode in DSR stems were measured. In rice stems, key genes involved in lignin biosynthesis and metabolism include *OsPAL*, *OsCOMT*, *Os4CL3*, *OsCAD2*, and *OsCAD7*. The expression of the *Os4CL3* culm of JYJ and JJ305 under soaking the seeds in paclobutrazol treatments was down-regulated, and the expression of the *OsPAL*, *OsCOMT*, *OsCAD2*, and *OsCAD7* culm of JYJ and JJ305 was up-regulated (Figure 10).

### 2.10. Principal Component Analysis (PCA)

**Figure 11:**
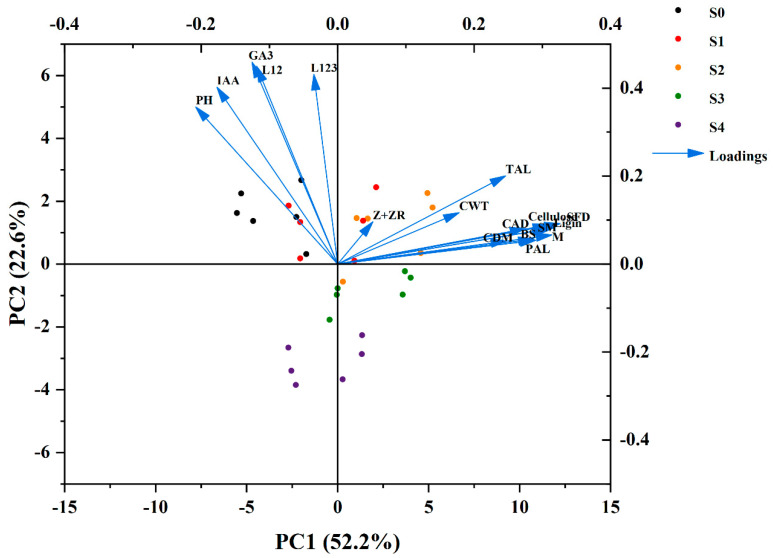
Principal component analysis (PCA) of breaking strength, hormone contents, morphological structure, and assimilate composition of the second basal internode of direct-seeded rice stem under seed soaking with paclobutrazol in the heading stage. Vectors illustrate trait factor loading coordinates for PC1 and PC2. M: breaking strength, SM: section modulus, BS: bending stress; IAA: indole acetic acid; GA_3_: gibberellin A3; Z+ZR: zeatin and zeatin riboside; L12: the percentage of the total internode length made up of basal internodes (I1 + I2); L123: the percentage of the total internode length made up of basal internodes (I1 + I2 + I3); CDM: culm diameter; CWT: culm wall thickness; CFD: the culm filling degree; PAL, phenylalanine ammonia-lyase activity; TAL, tyrosine ammonia-lyase activity; CAD, cinnamyl alcohol dehydrogenase activity.

The PCA of breaking strength, hormone contents, morphological structure, and assimilate composition showed clear differences among the different paclobutrazol concentrations in the two cultivars under seed soaking with paclobutrazol in the heading stage (Figure 11). Principal component 1 (PC1) and principal component 2 (PC2) accounted for 52.2 and 22.6% variability among the variables in Figure 11. A smaller acute angle (<90°) between loading vectors indicates a stronger correlation between variables. IAA and GA_3_ were clustered closer to the percentage of basal internodes (I1 + I2/I1 + I2 + I3) and were in the opposite direction of breaking strength (M, SM, BS) and assimilate composition (lignin, PAL, TAL, CAD, cellulose). Z+ZR were clustered closer to CDM, CWT, CFD, lignin, PAL, TAL, CAD, cellulose, SM, BS, and M. SM was clustered closer to M compared with BS. CAD was clustered closer to lignin compared with PAL and TAL (Figure 11).

### 2.11. The Relationships between the Culm Anatomical Characteristics and Endogenous Hormones, Morphological Characteristics

**Figure 12:**
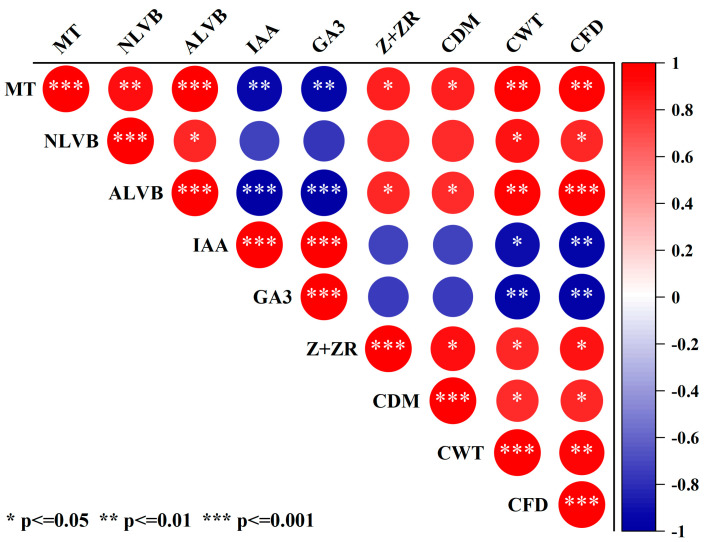
Relationship between the culm anatomical characteristics, endogenous hormones, and morphological characteristics of the second basal internode of direct-seeded rice. IAA: indole acetic acid; GA_3_: gibberellin A3; Z+ZR: zeatin and zeatin riboside; MT: mechanical tissue; NLVB: number of large vascular bundles; ALVB: area of large vascular bundles. CDM: culm diameter; CWT: culm wall thickness; CFD: the culm filling degree. The numbers in the figure represent r^2^; *, significant at 0.05 probability level. **, significant at 0.01 probability level. ***, significant at 0.001 probability level.

Pearson correlation was employed to analyze the relationships between the culm anatomical characteristics, endogenous hormones and morphological characteristics of the second basal internode in DSR. The culm wall thickness and filling degree were positively and significantly correlated with the mechanical tissue and number and area of large vascular bundles. The endogenous Z+ZR content was positively and significantly correlated with culm morphology (culm diameter, culm wall thickness, culm filling degree) and anatomical characteristics (the mechanical tissue and area of large vascular bundles) (Figure 12).

## 3. Discussion

Improving rice yield and quality is particularly urgent for ensuring China’s food security. In cereal crop production, stem lodging (stem bending or breaking) will limit yield and quality and reduce economic efficiency [13]. The poor lodging resistance of high-quality rice varieties is an important factor limiting the increase in its planting area [10]. Paclobutrazol is a plant growth regulator commonly used in production to regulate crop lodging. To our knowledge, previous studies have proven that paclobutrazol can regulate the growth and lodging resistance of crops such as corn [35], wheat [15], and rice [31]. Therefore, the application of paclobutrazol can be used as a technical way to control the lodging and yield formation of direct-seeded rice (DSR). The regulatory effect of paclobutrazol on crop growth is directly related to the growth stage of the crop, crop type, and application concentration [29]. At present, there is no systematic study on the performance of paclobutrazol on the yield and lodging resistance of DSR. Therefore, it is necessary to further analyze the changes in the morphological and anatomical structure of DSR internodes and the metabolic processes of cell wall components under paclobutrazol treatment, and to further elucidate the physiological mechanism by which paclobutrazol regulates the mechanical strength of the basal internode of DSR.

### 3.1. Paclobutrazol Increased the Yield and Decreased the Lodging Rate of Direct-Seeded Rice 

Studies have shown that paclobutrazol treatment can increase the crop yield. However, some studies have found that paclobutrazol can enhance the lodging resistance of crops but will not increase crop yield or even reduce crop yield [15,30,35]. The reason may be caused by the different application concentration and period of paclobutrazol [29]. The changes in the yield of direct-seeded rice (DSR) with the application method and concentration of paclobutrazol still need further research. In this study, soaking the seeds in paclobutrazol increased the yield of DSR, and the S2 treatment increased the yield of DSR compared to the other paclobutrazol treatments (Figure 1). Analyzing the reasons, on the one hand, it may be that paclobutrazol application can increase the yield of DSR by delaying leaf senescence and promoting biomass accumulation [36], thereby promoting yield formation. On the other hand, in this study, paclobutrazol application increased the number and area of big vascular bundles (Figure 9), where vascular bundles could provide transport and mechanical organization to the stem [19], resulting in more carbohydrate accumulation and transport [37], therefore enhancing stem strength and promoting the yield formation. In this study, further increasing the concentration of paclobutrazol did not further increase the yield, which is similar to the results of the study of Kamran et al. [35] on the effects of paclobutrazol seed soaking and seed dressing treatments on maize yield and lodging resistance in semi-arid areas. The above results show that, in this study, soaking seeds in paclobutrazol at concentrations of 100 mg L^−1^ can simultaneously improve the lodging resistance and yield of DSR.

### 3.2. Paclobutrazol Optimized the Morphological and Anatomical Structure of Direct-Seeded Rice Stems and Enhanced Lodging Resistance by Regulating the Endogenous Hormone Content

The stem of DSR above the ground consisted of nodes and internodes, which supported the plant, linked the leaves and panicles, and facilitated the transport of nutrients. Specifically, the basal internode of rice plants provides support for the heavier upper parts of the plant. Its growth not only impacts the production of yield but is also connected to resistance against lodging. The stiffness of the basal stem determines rice plant susceptibility to lodging [18]. Consequently, enhancing the physical strength of the basal stems has emerged as a new research target for enhancing crop lodging resistance and thus increasing grain yield. In this study, paclobutrazol application increased the breaking strength from 0d to 50d after the formation of the basal second internode, while the maximum breaking strength was obtained with the S2 treatment (Figure 5). The breaking strength can be further divided into two parts, namely section modulus (SM), which is affected by internode morphological traits (e.g., inner and outer diameter of culm, culm diameter, and wall thickness), and bending stress (BS), which is an indicator of the internode filling degree. Paclobutrazol application increased the SM and BS of the culm, suggesting that soaking the seeds in paclobutrazol can effectively regulate the structure and material composition of stems (Figure 5 and Figure 11). 

**Figure 13:**
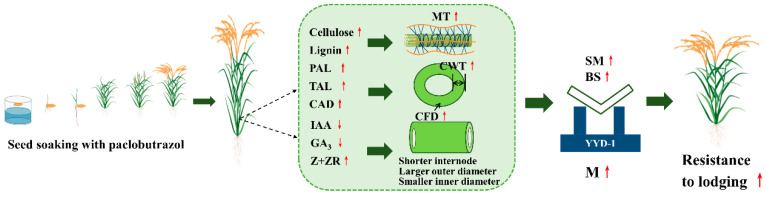
Analysis of the causes of lodging and ways to improve lodging resistance ability of DSR. M: breaking strength; SM: section modulus; BS: bending stress; IAA: indole acetic acid; GA_3_: gibberellin A3; Z+ZR: zeatin and zeatin riboside; CWT: culm wall thickness, CFD: the filling degree of the second basal internode; MT: mechanical tissue; PAL, phenylalanine ammonia-lyase activity; TAL, tyrosine ammonia-lyase activity; CAD, cinnamyl alcohol dehydrogenase activity.

Zhang et al. proposed that achieving optimal plant height through the reduction in basal internode length and the enhancement of peduncle length is beneficial for maximizing yield [38]. Internode length is closely related to internode mechanical strength. Shorter basal internodes may also enhance the mechanical strength of rice stems to some extent [28,39]. In this study, regarding paclobutrazol application, the percentage of the total internode length made up of basal internodes (I1 + I2 and I1 + I2 + I3) decreased (Figure 2C,D), and the magnitude of the decrease in internode length increased with the concentration of paclobutrazol. This finding was consistent with a report of wheat by [34]. Plant internode elongation is determined by cell morphology [27]. Plant hormones are crucial for regulating plant growth and development [40]. In wheat, the ratio of endogenous IAA/GA had significant effects on plant height by affecting cell length [41]. In maize, the high level of GA_3_ content in plants led to elongated and slender internodes, which increase the risk of fragility [28,42]. Paclobutrazol could affect the isoprenoid pathway, altering the levels of plant hormones by inhibiting gibberellin synthesis and increasing the cytokinin level, resulting in a reduction in stem elongation [29]. In this study, paclobutrazol application significantly reduced endogenous GA_3_ and IAA contents in the culm tissue of both cultivars (Figure 3), which is consistent with previous reports by [43]. The principal component analysis (PCA) found that stronger correlations were observed between the percentage of the total internode length made up of basal internodes (I1 + I2 and I1 + I2 + I3) and endogenous GA_3_, IAA contents (Figure 11). Therefore, paclobutrazol application reduced the cell length of the base second segment of DSR compared to the S0 treatment, thus shortening the basal internode length and optimizing stem structure of DSR (Figure 11 and Figure 13).

Morphological characteristics of the stem, including the diameter and wall thickness of the culm, are intimately associated with resistance to stem bending [16,28,44,45]. Thick basal internodes could contribute to enhancing the bending strength of plants [4,46]. In this study, culm diameter, culm wall thickness, and culm filling degree were significantly correlated with the breaking strength of the second basal internode (Figure 4). The application of paclobutrazol increased the culm diameter and culm wall thickness (Figure 4A,B). The range of increase in culm wall thickness was significantly greater than that in culm diameter, suggesting that enhancing the culm wall thickness was more effective than increasing the culm diameter for improving lodging resistance in DSR [28]. These findings could be explained as follows. First, studies indicated that high-quality stems have thick stalk walls and moderate pulp cavity sizes [47]. In this study, as paclobutrazol application increased, the outer diameter increased but the inner diameter decreased, resulting in the culm cross-sectional area increasing rapidly and the pith area decreasing [48]. Second, Z+ZR is a biologically important cytokinin in rice plants [49]. The endogenous Z content increased significantly when paclobutrazol was applied to both varieties (Figure 3). Endogenous Z could cause the cells to become smaller and more densely packed. These results distinctly demonstrate that the application of paclobutrazol increased the endogenous Z content in DSR plants, leading to the horizontal expansion and vertical division of cells in culm tissue, similar to the report by Cui et al. (1997) [41] and Lv et al. (2022) [28]. In addition, the mechanical strength is determined by the thickness of the mechanical tissue and the vascular bundle sheath, and enhancing the thickness of the mechanical tissue layer is the most effective way to boost the lodging resistance of rice at the tissue level [21]. A thicker mechanical tissue with densely arranged thick-walled cells and vascular bundle sheath cells results in higher mechanical strength [11,24]. This study indicated a significant increase in mechanical tissue thickness and number and area of large vascular bundles under paclobutrazol treatment (Figure 9). These anatomical changes explain why paclobutrazol treatment enhances the mechanical strength of the basal internode. Correlation analysis revealed that the content of the endogenous Z+ZR of the culm was closely linked to mechanical tissue thickness and vascular bundle development (Figure 12). The mechanical tissue thickness and number and area of large vascular bundles were closely linked to culm diameter, culm wall thickness, and culm filling degree (Figure 12). These results suggest that paclobutrazol treatment promoted morphological and anatomical characteristics of the culm to increase the section modulus by regulating endogenous hormone levels. This ultimately enhanced the mechanical strength of the stem (Figure 13).

### 3.3. Paclobutrazol Promoted the Development of Cell Walls through Internode Carbohydrate Anabolism, Thereby Enhancing Stem Lodging Resistance

Previous studies have revealed that the culm plumpness of the basal internode has a positive impact on the stem mechanical strength in cereal crops [17]. In this study, paclobutrazol application significantly increased the culm filling degree of the basal internode in DSR (Figure 4C), aligning with the results of previous studies in maize [23] and winter wheat [34]. There is a significant positive correlation among stem plumpness and structural carbohydrates (cellulose and lignin) and nonstructural carbohydrates (starch and soluble) in rice plants [50]. Cellulose and lignin are synthesized and deposited as a strong fibrillary network in the sclerenchyma cells of mechanical tissue and vascular bundle sheaths [28,51]. Consequently, there is a notable connection between cellulose, lignin, and the stem strength in cereal plants [10]. In the current study, paclobutrazol application significantly increased the culm lignin and cellulose contents of the second basal internode in both varieties and years (Figure 6 and Figure 8). The PCA found that lignin and cellulose contents were positively correlated with the breaking strength (Figure 11). This suggests that the paclobutrazol application enhanced the bending stress value by increasing the lignin and cellulose of the second basal internode in DSR, partly enhancing the stem strength and thus reducing the occurrence of lodging (Figure 13).

Lignin, a complex aromatic biopolymer, is mainly deposited in secondary thickened cell walls through cross-linking with cellulose and hemicellulose, increasing its rigidity [52]. This provides structural support to the wall and assists in the transport of water and nutrients within xylem tissue by reducing the permeability of the cell wall [53]. Currently, lignin content at the basal internode has been recognized as a significant measure of lodging resistance in crops. The activities of the CAD, PAL, POD, TAL, and 4CL enzymes play key roles in lignin biosynthesis [54,55]. Ahmad et al. reported that paclobutrazol significantly increased the activities of maize PAL, CAD, TAL, and POD enzymes [23]. Our study shows that paclobutrazol application improved the activity of PAL, TAL, and CAD enzymes (Figure 7), promoted the accumulation of lignin (Figure 6), and improved the internode mechanical strength of DSR throughout the internode formation period (Figure 11 and Figure 13). Similar findings indicate that the accumulation of lignin had a significant positive correlation with the enzyme activity of PAL, 4CL, CAD, and POD, as well as with M [22]. The PCA found that CAD was clustered closer to lignin compared with PAL and TAL (Figure 11), suggesting that lignin accumulation more closely correlated with the enzyme activity of CAD [56]. Further analysis of key genes in lignin biosynthesis showed that soaking the seeds in paclobutrazol up-regulated the expression of the *OsPAL*, *OsCOMT*, *OsCAD2*, and *OsCAD7* culm of DSR (Figure 10). These results show that soaking the seeds in paclobutrazol promoted lignin biosynthesis by enhancing the expression of key genes in lignin biosynthesis, thus enhancing the mechanical strength of the stem.

## 4. Materials and Methods

### 4.1. Experimental Location

**Figure 14:**
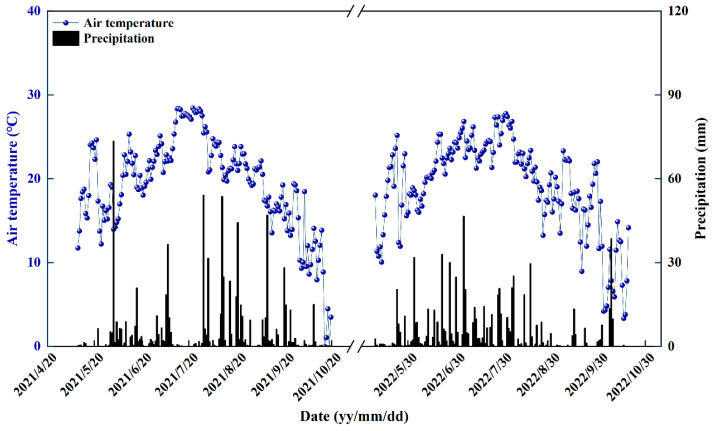
Daily average temperature and precipitation during the whole rice growth stage in 2021–2022. The columns illustrate the precipitation and the plotted lines represent the daily average temperature.

This study was conducted in 2021 and 2022 at the National Crop Variety Validation Characterization Station on the campus of Jilin Agricultural University in Changchun, Jilin Province, China (43°81 N, 125°42 E, 221 m in altitude). Figure 14 illustrates the daily precipitation and temperature during the experimental period. This information was gathered from an automated weather station situated at the experimental site. The soil properties of the experimental site were as follows: chernozem with 12.06 g kg^−1^ organic carbon, 47.43 mg kg^−1^ alkaline hydrolysis N, 10.72 mg kg^−1^ available P, 130.24 mg kg^−1^ available K, and pH 6.3 (H_2_O).

### 4.2. Field Experiment, Materials, and Design

The experiment was a randomized complete block design with three replications. Each plot covered an area of 30 m^2^ (5 m × 6 m). All plots were separated by bunds and had a separate draining outlet to drain water into the ditches.

The japonica rice varieties Jiyujing (JYJ) and Jijing305 (JJ305), which are mainly used for rice production in Jilin Province, were selected as experimental materials. The crop durations from sowing to maturity of JYJ and JJ305 were 135 d and 134 d, respectively. Rice seeds were sterilized using a 0.3% sodium hypochlorite solution for 10 min, followed by three washes with distilled water. The rice seeds were then soaked in paclobutrazol solution at concentrations of 0 (S0), 50 (S1), 100 (S2), 150 (S3), and 200 (S4) mg L^−1^ of seeds at room temperature for 24 h in an incubator. Seeds were removed from solutions and air-dried at room temperature prior to sowing. The plant growth regulator paclobutrazol was provided by Sichuan Guoguang Agrochemical Co., Ltd., Chengdu, Sichuan, China. 

The row-to-row spacing was 25 cm, and the plant-to-plant spacing was 13.3 cm. Seeds were sown on 5 and 6 May and harvested on 5 and 7 October in 2021 and 2022, respectively. Nitrogen was applied in the form of urea at a rate of 150 kg ha^−1^, phosphorus was applied as single superphosphate at a rate of 75 kg ha^−1^, and potassium was applied as potassium sulfate at a rate of 75 kg ha^−1^. Fertilizer N was applied three times as 40% base fertilizer before seeding and at the early tillering (30%) and panicle initiation (30%) stages. Fertilizer K_2_O was split evenly between basal application (50%) and panicle initiation (50%). Fertilizer P_2_O_5_ was applied as a basal fertilizer. In all plots, pre-emergence herbicides and hand weeding were employed for controlling weeds. Pesticides and fungicides were sprayed to control pests and diseases if needed. 

### 4.3. Measuring Items and Method

#### 4.3.1. Yield and Lodging Rate

At maturity, the rice grains collected from 5 m^2^ blocks for each treatment were used to measure grain yield by moisture content of 14%. Lodging rate was investigated using the methods described by Peng et al. [15]. The lodging area for each plot was quantified, and the lodging percentage was calculated using the formula: lodging rate (%) = (the lodging area in plot/the plot area) ×100.

#### 4.3.2. Morphological Characteristics of the Basal Second Internode

At the beginning of the elongation stage of rice, 300 uniform stems per plot were tagged with red thread. There were five elongated internodes on the main stems of JYJ and JJ305, which are referred to as the 1st (I1), 2nd (I2), 3rd (I3), 4th (I4), and 5th (I5) internodes, which are numbers from the bottom of the culm. At heading stage, the length of each internode was measured using a ruler. Morphological characteristics of the basal second internode were performed after the formation of the second basal internode in DSR stems. Ten representative main stems were gathered at 0d, 10d, 20d, 30d, 40d, and 50d after the formation of the second basal internode (DAF0d, DAF10d, DAF20d, DAF30d, DAF40d, and DAF50d), and there was a total of six collections. The inner and outer diameter and wall thickness of the second basal internode were measured with a digital Vernier caliper with an accuracy of 0.01 mm. After the leaf sheath of the stem internode was removed, the culms were oven-dried to constant weight, and dry weight was determined. The filling degree of the culm was scored with the following formulas:

Culm filling degree = the dry weight of the second basal culm/length of the basal second culm. (1)

#### 4.3.3. Determination of Endogenous Hormones

Five fresh representative main stems were sampled from each plot to determine the stem endogenous hormones at the second leaf stage (the second basal internode of the DSR stem undergoes rapid elongation). The culm of the second basal internode was ground using liquid nitrogen. The extraction and analytical conditions of indole acetic acid (IAA), gibberellin A3 (GA_3_), and zeatin and zeatin riboside (Z+ZR) were modified based on previous research (Wang et al., 2020; Glauser et al., 2016). ESI high-performance liquid chromatography–tandem mass spectrometry (ESI-HPLC-MS/MS) (Agilent 1290, Agilent, Santa Clara, CA, USA; 6500 Qtrap, AB Sciex, Framingham, MA, USA) was used for determination of hormone contents. Column: Poroshell 120 SB-C18 reversed-phase column (2.1 × 150, 2.7 μm); column temperature: 30 °C; mobile phase: A: B = (methanol/0.1% formic acid): (water/0.1% formic acid); elution gradient: 0–1 min, A = 20%; 1–9 min, A incremented to 80%; 9–10 min, A = 80%; 10-A = 20% for 10.1 min and A = 20% for 10.1–15 min; injection volume: 2 µL. Mass spectrometry data were determined using Analyst 1.6.2 software.

#### 4.3.4. Determination of the Breaking Strength of the Second Basal Internode

At 0d, 10d, 20d, 30d, 40d, and 50d after the formation of the second basal internode (DAF0d, DAF10d, DAF20d, DAF30d, DAF40d, and DAF50d), the breaking strength of the second basal internode with the leaf sheath was determined using a stem strength tester (YYD-1, Hangzhou TOP Instrument Co., Ltd., Hangzhou, China) according to the method reported by Ookawa and Ishihara [57]. Physical parameters were calculated as follows [1,57,58,59].

(1) Breaking strength (M, g cm), M = BL × L × 1/4 × 10^3^, where BL is the force applied to break the stem segment (kg) and L is the distance between two points (cm).
(2) Section modulus (SM, mm^3^): SM = π/32 × (a_1_^3^b_1_ – a_2_^3^b_2_)/a_1,_ where b_1_ is the outer diameter of the major axis in an oval cross-section (mm), b_2_ is inner diameter of the major axis in an oval cross-section (mm), a_1_ is outer diameter of the minor axis in an oval cross-section (mm), and a_2_ is inner diameter of the minor axis in an oval cross-section (mm).
(3) Bending stress (BS, g mm^−2^): BS = M×10/SM.

#### 4.3.5. Lignin Determination

After the formation of the basal second internode, 10 labeled stems from each plot were taken every 10 days. The sampled basal second internode with the removed stem sheath was immediately treated with liquid nitrogen and subsequently stored at −80 °C for determining lignin accumulation, related enzyme activities, and expression of genes involved in the lignin synthesis.

##### Lignin Content

The determination of lignin content was performed according to the method described [15,60]. A total of 300 mg of freshly homogenized stem samples was ground into powder in liquid nitrogen, with 80% ethanol for 2 h to remove soluble metabolites, followed by a 1 h extraction in chloroform at 62 °C, and dried at 50 °C. Subsequently, the dried sediments were digested in a 25% (*v*/*v*) solution of bromoacetyl (containing 2.7% (*v*/*v*) perchloric acid) at 70 °C for 1 h. The samples were cooled to room temperature. A total of 0.3 mL of each sample was added to 1.9 mL of 2 mol L-1 NaOH, 0.1 mL of 7.5 mol L^−1^ hydroxylamine hydrochloride, and acetic acid to ensure termination of the reaction. The volume was corrected to 5 mL with acetic acid, and the OD value at a wavelength of 280 nm was measured using a spectrophotometer (Shimadzu UV-2450, Tokyo, Japan). Lignin was expressed by OD280 mL^−1^ g^−1^ FW.

##### Enzyme Extraction and Assays

The activities of phenylalanine ammonia-lyase (PAL) and tyrosine ammonia-lyase (TAL) were extracted and assayed in accordance with the method described by Assis et al. [61], and the activity of cinnamyl alcohol dehydrogenase (CAD) was extracted and assayed by adapting the procedure used by Morrison et al. [62], with slight modifications.

##### Expression of Genes Involved in the Synthesis of Lignin

To extract the total RNA of the culm tissues of the second basal internode of the DSR plant, a *TransZol* Plant RNA extraction kit (Beijing, China) was utilized following the manufacturer’s instructions. The synthesis of the first-strand cDNA was performed using a *TransScript*^®^ All-in-One First-Strand cDNA Synthesis Super Mix for qPCR (One-Step gDNA Removal) (Takara, Japan) in accordance with the manufacturer’s instructions. Gene expression was tested in three replicates by quantitative polymerase chain reaction (qPCR) with *Trans PerfectStart*^®^ Green qPCR Super Mix Fluorescence Quantitative Kit according to the manufacturer’s instructions with the following cycling profile: denaturation, 94 °C for 30 s, 1 cycle; PCR, 94 °C for 5 s and 60 °C for 30 s, 40 cycles; melting, 95 °C for 5 s (4.4 °C/s/L cooling rate) and 60 °C for 1 min (2.2 °C/s/L cooling rate), 95 °C (0.11 °C/s/L cooling rate; continuous acquisition mode, 5 acquisitions per °C), 1 cycle. The primers sequences used in this study are shown in Table 1.

#### 4.3.6. Determination of Cellulose Content

The samples of the basal second internode were collected at 0d, 10d, 20d, 30d, 40d, and 50d after the formation of the basal second internode (DAF0d, DAF10d, DAF20d, DAF30d, DAF40d, and DAF50d). The dry weight of each sample was then determined after oven-drying at 80 °C to constant weight. Subsequently, the samples were ground into a fine powder. The cellulose content was measured in accordance with the modified procedure by the studies described [1,63].

#### 4.3.7. Microstructure of the Basal Second Internode of Stem

Five fresh representative main stems were selected in each plot at the heading stage, and stem segments of 2 cm in the middle of the second basal internode were promptly cut using a double-sided blade and fixed with FAA (38% formaldehyde: 70% glacial acetic acid: ethanol, 1:1:18) fixation solution. The images of the prepared slices were collected using a slice scanner and analyzed with the CaseViewer 2.4.0 slice scanning software. The software was employed to measure the mechanical tissue, the number of large vascular bundles, and the area of large vascular bundles [63,64].

### 4.4. Statistical Analysis

The statistical analyses were performed at the 0.05 level of significance. Table and figures were prepared in Microsoft Excel 2021 software for Windows. For multiple comparison tests, the comparison of means was analyzed using Tukey’s test at *p* < 0.05 with SPSS 16.0. Correlations between different parameters were conducted with Pearson’s correlation in SPSS. Origin 23.0 software (Origin Lab, Northampton, MA, USA) was used to plot the data. Principal component analysis (PCA) of the breaking strength and morphological traits, anatomical characteristics, carbohydrate content, and endogenous hormones were analyzed by Origin 23.0. 

## 5. Conclusions

Soaking the seeds in paclobutrazol optimized the morphological and anatomical structure of the internodes in direct-seeded rice by regulating the endogenous IAA, GA_3_, and Z+ZR contents and promoted the development of internode cell walls by promoting internodal carbohydrate anabolism, thereby strengthening the breaking strength of the stem. Under the experiment conditions, the effect was the most significant when the seed soaking concentration of paclobutrazol was 100 mg L^−1^. At the time, the lodging rate of DSR decreased by 13.8% and the yield of DSR increased by 14.0% compared with the control treatment. Scientific chemical regulation can enhance the lodging resistance of DSR and facilitate achieving a high and stable yield.

## Figures and Tables

**Table 1:** List of primer sets used for quantitative RT-PCR.

Gene	Forward Primer (5′-3′)	Reverse Primer (5′-3′)
*Os4CL3*	GCCGTCTCCTCGTGTAAC	TTGGCCTTAGCTGCTTTT
*OsCAD2*	CGACCAGAAGTTTGTGGTGAA	GAAGTGCTTCAGTGGGCTGTA
*OsCAD7*	TCACCGGGGTGGTGACCGAG	CCGCCGCAGGTGTTCACCAT
*OsPAL*	ACCGCTTCGTGTATCTTCAG	AAGGATGGAATCGAGTAGCA
*OsCOMT*	GAAGGTGGTGGTGGTGGAGT	GCGTTGGCGTAGATGTAGGTG
*OsActin*	CAATCGTGAGAAGATGACCC	GTCCATCAGGAAGCTCGTAGC

Accession numbers are XM_015770230 (*Os4CL3*), XM_015770922 (*OsCAD2*), XM_015780124 (*OsCAD7*), XM_015769634 (*OsPAL*), XM_015794567 (*OsCoMT*), and XM_015774830 (*OsActin*).

## Data Availability

Data will be made available on request.

## References

[1] Liu W.Y., Fan X.H., Liu Y.Y., Bao S.Y., Lu Y.Y., Gai D.S., Fu X.K., Du J., Guo L.Y., Zhang Q. (2023). Relationship between characteristics of basal internodes and lodging and its physiological mechanism in direct-seeded rice. J. Agron. Crop Sci..

[2] Kobayashi K., Wang X., Wang W. (2023). Genetically Modified Rice Is Associated with Hunger, Health, and Climate Resilience. Foods.

[3] Liang C., Li Y., Zhang K., Wu Z., Liu J., Liu J., Zhou C., Wang S., Li F., Sui G. (2023). Selection and Yield Formation Characteristics of Dry Direct Seeding Rice in Northeast China. Plants.

[4] Farooq M., Siddique K.H.M., Rehman H., Aziz T., Lee D.J., Wahid A. (2011). Rice direct seeding: Experiences, challenges and opportunities. Soil Till. Res..

[5] Liu H., Hussain S., Zheng M., Peng S., Huang J., Cui K., Nie L. (2015). Dry direct-seeded rice as an alternative to transplanted-flooded rice in Central China. Agron. Sustain. Dev..

[6] Wang W.Q., Peng S.B., Liu H.Y., Tao Y., Huang J.L., Cui K.H., Nie L.X. (2017). The possibility of replacing puddled transplanted flooded rice with dry seeded rice in central China: A review. Field Crops Res..

[7] Rao A.N., Nagamani A. Available technologies and future research challenges for managing weeds in dry-seeded rice in India. Proceedings of the 21st Asian Pacific Weed Science Society (APWSS) Conference.

[8] Gianessi L., Silvers C., Sankula S., Carpenter J. (2002). Plant Biotechnology: Current and Potential Impact for Im-proving Pest Management in U.S. Agriculture: Case Study 27, Herbicide Tolerant Rice.

[9] Pandey S., Velasco L. (2002). Economics of direct seeding in Asia: Patterns of adoption and research priorities. Direct Seeding: Research Strategies and Opportunities.

[10] Niu Y.N., Chen T.X., Zhao C.C., Zhou M.X. (2022). Lodging prevention in cereals: Morphological, biochemical, anatomical traits and their molecular mechanisms, management and breeding strategies. Field Crop. Res..

[11] Khobra R., Sareen S., Meena B.K., Kumar A., Tiwari V., Singh G.P. (2019). Exploring the traits for lodging tolerance in wheat genotypes: A review. Physiol. Mol. Biol. Plants.

[12] Zhang R., Jia Z.F., Ma X., Ma H.L., Zhao Y.W. (2020). Characterising the morphological characters and carbohydrate metabolism of oat culms and their association with lodging resistance. Plant Biol..

[13] Lang Y.Z., Yang X.D., Wang M.E., Zhu Q.S. (2012). Effects of lodging at different filling stages on rice yield and grain quality. Rice Sci..

[14] Xing Z.P., Wu P., Zhu M., Qian H.J., Cao W.W., Hu Y.J., Guo B.W., Wei H.Y., Xu K., Dai Q.G. (2017). Effect of mechanized planting methods on plant type and lodging resistance of different rice varieties. Trans. Chin. Soc. Agric. Eng..

[15] Peng D.L., Chen X.G., Yin Y.P., Lu K.L., Yang W.B., Tang Y.H., Wang Z.L. (2014). Lodging resistance of winter wheat (*Triticum aestivum* L.): Lignin accumulation and its related enzymes activities due to the application of paclobutrazol or gibberellin acid. Field Crops Res..

[16] Zhang W.J., Yao X., Duan X.J., Liu Q.M., Tang Y.Q., Li J.Y., Li G.H., Ding Y.F., Liu Z.H. (2021). Foliar application uniconazole enhanced lodging resistance of hybrid indica rice by altering basal stem quality under poor light stress. Agron. J..

[17] Dong X.C., Qian T.F., Chu J.P., Zhang X., Liu Y.J., Dai X.L., He M.R. (2023). Late sowing enhances lodging resistance of wheat plant via improving biosynthesis and accumulation of lignin and cellulose. J. Integr. Agric..

[18] Yang H., Huang J., Ye Y., Xu Y., Xiao Y., Chen Z., Li X., Ma Y., Lu T., Rao Y. (2024). Research Progress on Mechanical Strength of Rice Stalks. Plants.

[19] Shah A.N., Tanveer M., Rehman A.U., Anjum S.A., Iqbal J., Ahmad R. (2017). Lodging stress in cereal-effects and management: An overview. Environ. Sci. Pollut. Res..

[20] Wang X.Y., Le X., LI X.X., Yang G.D., Wang F., Peng S.B. (2022). Grain yield and lodging-related traits of ultrashort-duration varieties for direct-seeded and double-season rice in Central China. J. Integr. Agric..

[21] Xu S., Zhang M., Ye J., Hu D., Zhang Y., Li Z., Liu J., Sun Y., Wang S., Yuan X. (2023). Brittle culm 25, which encodes anUDP-xylose synthase, affects cell wall properties in rice. Crop. J..

[22] Wang C., Hu D., Liu X.B., She H.Z., Ruan R.W., Yang H., Yi Z.L., Wu D.Q. (2015). Effects of uniconazole on the lignin metabolism and lodging resistance of culm in common buckwheat (*Fagopyrum esculentum* M.). Field Crop. Res..

[23] Ahmad I., Kamran M., Ali S., Bilegjargal B., Cai T., Ahmad S., Meng X.P., Su W.N., Liu T.N., Han Q.F. (2018). Uniconazole application strategies to improve lignin biosynthesis, lodging resistance and production of maize in semiarid regions. Field Crop Res..

[24] Wang W.X., Du J., Zhou Y.Z., Zeng Y.J., Tan X.M., Pan X.H., Shi Q.H., Wu Z.M., Zeng Y.H. (2021). Effects of different mechanical direct seeding methods on grain yield and lodging resistance of early indica rice in South China. J. Integr. Agric..

[25] Huber H., Brouwer J., Wettberg E.J., During H.J., Anten N.P.R. (2013). More cells, bigger cells or simply reorganization? Alternative mechanisms leading to changed internode architecture under contrasting stress regimes. New Phytol..

[26] Zhang W., Wu L., Wu X., Ding Y., Li G., Li J., Weng F., Liu Z., Tang S., Ding C. (2016). Lodging resistance of Japonica rice (*Oryza sativa* L.): Morphological and anatomical traits due to top-dressing nitrogen application rates. Rice.

[27] Fang X.M., Liu X., Zhang Y.K., Huang K.H., Li Y.S., Nie J., She H.Z., Ruan R.W., Yuan X.H., Yi Z.L. (2018). Effects of uniconazole or gibberellic acid application on the lignin metabolism in relation to lodging resistance of culm in common buckwheat (*Fagopyrum esculentum* M.). J. Agron. Crop Sci..

[28] Lv R.J., Zhang W.J., Xie X.B., Wang Q.J., Gao K.G., Zeng Y.H., Zeng Y.J., Pan X.H., Shang Q.Y. (2022). Foliar application uniconazole enhanced lodging resistance of high-quality indica rice (*Oryza sativa* L.) by altering anatomical traits, cell structure and endogenous hormones. Field Crops Res..

[29] Desta B., Amare G. (2021). Paclobutrazol as a plant growth regulator. Chem. Biol. Technol. Agric..

[30] Rady M., Gaballah S. (2012). Improving barley yield grown under water stress conditions. Res. J. Recent. Sci..

[31] Xing P.P., Duan M.Y., Liu Y.H., Mo Z.W., Lai R.F., Tang X.R. (2022). Enhancement of Yield, Grain Quality Characters, 2-Acetyl-1-Pyrroline Content, and Photosynthesis of Fragrant Rice Cultivars by Foliar Application of Paclobutrazol. Plant Growth Regul..

[32] Zhao J.H., Lai H.J., Bi C., Zhao M.J., Liu Y.L., Li X.D., Yang D.Q. (2023). Effects of paclobutrazol application on plant architecture, lodging resistance, photosynthetic characteristics, and peanut yield at different single-seed precise sowing densities. Crop J..

[33] Kamran M., Ahmad S., Ahmad I., Hussain I., Meng X.P., Zhang X.D., Javed T., Ullah M., Ding R.X., Xu P.Z. (2020). Paclobutrazol application favors yield improvement of maize under semiarid regions by delaying leaf senescence and regulating photosynthetic capacity and antioxidant system during grain-filling stage. Agronomy.

[34] Kamran M., Ahmad I., Wu X.R., Liu T.N., Ding R.X., Han Q.F. (2018). Application of paclobutrazol: A strategy for inducing lodging resistance of wheat through mediation of plant height, stem physical strength, and lignin biosynthesis. Environ. Sci. Pollut. Res..

[35] Kamran M., Wennan S., Ahmad I., Meng X.P., Cui W.W., Zhang X.D., Mou S.W., Khan A., Han Q.F., Liu T.N. (2018). Application of paclobutrazol affect maize grain yield by regulating root morphological and physiological characteristics under a semi-arid region. Sci. Rep..

[36] Gai D.S., Liu W.Y., Liang J.N., Guo L.Y., Geng Y.Q., Zhang Q., Du J., Gao J.Q., Shao X.W. (2023). The Effects of Paclobutrazol Seed Soaking on Biomass Production and Yield Formation in Direct-Seeded Rice. Agronomy.

[37] Khanna V.K. (1991). Relationship of lodging resistance yield to anatomical characters of stem in wheat [*Triticum* spp.], triticale and rye. Wheat. Inf. Serv..

[38] Zhang M.C., Liu Y.Y., Luo S.G., Peng X.L., Chen L.N., Li Z.Y., Li J. (2010). Effects of integrated nutrient management on lodging resistance of rice in cold area. Sci. Agric. Sin..

[39] Zhong X.H., Liang K.M., Peng B.L., Tian K., Li X.J., Huang N.R., Liu Y.Z., Pan J.F. (2019). Basal internode elongation of rice as affected by light intensity and leaf area. Crop J..

[40] Ahmad I., Kamran M., Meng X.P., Ali S., Ahmad S., Gao Z.Q., Liu T.N., Han Q.F. (2021). Hormonal changes with uniconazole trigger canopy apparent photosynthesis and grain filling in wheat crop in a semi-arid climate. Protoplasma.

[41] Cui S.X., Huang H.Y., Wang D. (1997). Uniconazole induced morphological change and its relation to endogenous hormones in wheat seedings. Acta Bot. Boreali-Occident. Sin..

[42] Voorend W., Nelissen H., Vanholme R., Vliegher A.D., Breusegem F.V., Boerjan W., Rold’an-Ruiz I., Muylle H., Inz’e D. (2016). Overexpression of GA20-OXIDASE1 impacts plant height, biomass allocation and saccharification efficiency in maize. Plant Biotechnol. J..

[43] Wang X., Yu M.Y., Tao L.X., Huang X.L. (1997). Effect of pentefezole on the endogenous IAA content in rice seedings. Acta Bot. Sin..

[44] Li C.H., Li W.Q., Long Y.L., Jin M., Chang Y.L., Cui H.X., Sun S.F., Li Y., Wang Z.L. (2023). Mixed cropping increases grain yield and lodging resistance by improving the canopy light environment of wheat populations. Eur. J. Agron..

[45] Li L., He L.X., Li Y.X., Wang Y.F., Ashraf U., Hamoud U.A., Hu X., Wu T.Y., Tang X.R., Pan X.G. (2023). Deep fertilization combined with straw incorporation improved rice lodging resistance and soil properties of paddy fields. Eur. J. Agron..

[46] Liu C., Zheng S., Gui J.S., Fu C.J., Yu H.S., Song D.L., Shen J.H., Qin P., Liu X.M., Han B. (2018). Shortened basal internodes encodes a Gibberellin 2-Oxidase and contributes to lodging resistance in rice. Mol. Plant..

[47] Li W.Q., Han M.M., Pang D.W., Chen J., Wang Y.Y., Dong H.H., Chang Y.L., Jin M., Luo Y.L., Li Y. (2022). Characteristics of lodging resistance of high-yield winter wheat as affected by nitrogen rate and irrigation managements. J. Integr. Agric..

[48] Yin X., Tao Z., Huang M., Zou Y. (2018). Increasing wall thickness is more effective than increasing diameter for improving breaking resistance of rice internode. J. Plant Biol. Crop Res..

[49] Zhang W.Y., Cao Z.Q., Zhou Q., Chen J., Xu G.W., Gu J.F., Liu L.J., Wang Z.Q., Yang J.C., Zhang H. (2016). Grain filling characteristics and their relations with endogenous hormones in large- and small-grain mutants of rice. PLoS ONE.

[50] Zhu M.C., Lin C.H., Jiang Z.R., Yan F.Y., Li Z.Y., Tang X.N., Yang F., Ding Y.F., Li W.W., Liu Z.H. (2023). Uniconazole enhances lodging resistance by increasing structural carbohydrate and sclerenchyma cell wall thickness of japonica rice (*Oryza sativa* L.) under shading stress. Environ. Exp. Bot..

[51] Zhang W.J., Wu L.M., Ding Y.F., Yao X., Wu X.R., Weng F., Li G.H., Liu Z.H., Tang S., Ding C.Q. (2017). Nitrogen fertilizer application affects lodging resistance by altering secondary cell wall synthesis in japonica rice (*Oryza sativa* L.). J. Plant Res..

[52] Chen X.G., Shi C.Y., Yin Y.P., Wang Z.L., Shi Y.H., Peng D.L., Ni Y.L., Cai T. (2011). Relationship between lignin metabolism and lodging resistance in wheat. Acta Agron. Sin..

[53] Boerjan W., Ralph J., Baucher M. (2003). Lignin biosynthesis. Annu. Rev. Plant Biol..

[54] Fetherolf M.M., Levy-Booth D.J., Navas L.E., Liu J., Grigg J.C., Wilson A., Katahira R., Beckham G.T., Mohn W.W., Eltis L.D. (2020). Characterization of alkylguaiacol-degrading cytochromes P450 for the biocatalytic valorization of lignin. Proc. Natl. Acad. Sci. USA.

[55] Fang L., Xu X., Li J., Zheng F., Li M.Z., Yan J.W., Li Y., Zhang X.H., Li L., Ma G.H. (2020). Transcriptome analysis provides insights into the non-methylated lignin synthesis in paphiopedilum armeniacum seed. BMC Genom..

[56] Boudet A.M., Kajita S., Grima-Pettenati J., Goffner D. (2003). Lignins and lignocellulosic-s: A better control of synthesis for new and improved uses. Trends Plant Sci..

[57] Ookawa T., Ishihara K. (1992). Varietal difference of physical characteristics of the culm related to lodging resistance in paddy rice. Jpn. J. Crop Sci..

[58] Islam M.S., Peng S.B., Visperas R.M., Ereful N., Bhuiya M.S.U., Julfiquar A.W. (2007). Lodging-related morphological traits of hybrid rice in a tropical irrigated ecosystem. Field Crops Res..

[59] Ookawa T., Hobo T., Yano M., Murata K., Ando T., Miura H., Asano K., Ochiai Y., Ikeda M., Nishitani R. (2010). New approach for rice improvement using a pleiotropic QTL gene for lodging resistance and yield. Nat. Commun..

[60] Syros T., Yupsanis T., Zafiriadis H., Economou A. (2004). Activity and isoforms of peroxidases, lignin and anatomy, during adventitious rooting in cuttings of *Ebenus cretica* L.. J. Plant Physiol..

[61] Assis J.S., Maldonado R., Muñoz T., Escribano M.I., Merodio C. (2001). Effect of high carbon dioxide concentration on PAL activity and phenolic contents in ripening cherimoya fruit. Postharvest Biol. Technol..

[62] Morrison T.A., Kessler J.R., Hatfield R.D., Buxton D.R. (1994). Activity of two lignin biosynthesis enzymes during development of a maize internode. J. Sci. Food Agric..

[63] Updegraff D.M. (1969). Semimicro determination of cellulose in biological materials. Anal. Biochem..

[64] Wang Y.Y., Jin M., Luo Y.L., Chang Y.L., Zhu J.K., Li Y., Wang Z.L. (2022). Effects of irrigation on stem lignin and breaking strength of winter wheat with different planting densities. Field Crops Res..

